# The New Microtubule-Targeting Agent SIX2G Induces Immunogenic Cell Death in Multiple Myeloma

**DOI:** 10.3390/ijms231810222

**Published:** 2022-09-06

**Authors:** Katia Grillone, Caterina Riillo, Roberta Rocca, Serena Ascrizzi, Virginia Spanò, Francesca Scionti, Nicoletta Polerà, Annalisa Maruca, Marilia Barreca, Giada Juli, Mariamena Arbitrio, Maria Teresa Di Martino, Daniele Caracciolo, Pierosandro Tagliaferri, Stefano Alcaro, Alessandra Montalbano, Paola Barraja, Pierfrancesco Tassone

**Affiliations:** 1 Department of Experimental and Clinical Medicine, Magna Graecia University of Catanzaro, 88100 Catanzaro, Italy; 2 Net4Science s.r.l., Academic Spinoff, Magna Graecia University of Catanzaro, 88100 Catanzaro, Italy; 3 Department of Biological, Chemical and Pharmaceutical Sciences and Technologies (STEBICEF), University of Palermo, 90128 Palermo, Italy; 4 Institute of Research and Biomedical Innovation (IRIB), Italian National Council (CNR), 98122 Messina, Italy; 5 Institute of Research and Biomedical Innovation (IRIB), Italian National Council (CNR), 88100 Catanzaro, Italy; 6 Department of Health Sciences, Magna Graecia University of Catanzaro, 88100 Catanzaro, Italy

**Keywords:** cancer treatment, immunogenic cell death, ICD inducers, MTAs, multiple myeloma

## Abstract

Microtubule-targeting agents (MTAs) are effective drugs for cancer treatment. A novel diaryl [1,2]oxazole class of compounds binding the colchicine site was synthesized as cis-restricted-combretastatin-A-4-analogue and then chemically modified to have improved solubility and a wider therapeutic index as compared to vinca alkaloids and taxanes. On these bases, a new class of tricyclic compounds, containing the [1,2]oxazole ring and an isoindole moiety, has been synthetized, among which SIX2G emerged as improved MTA. Several findings highlighted the ability of some chemotherapeutics to induce immunogenic cell death (ICD), which is defined by the cell surface translocation of Calreticulin (CALR) via dissociation of the PP1/GADD34 complex. In this regard, we computationally predicted the ability of SIX2G to induce CALR exposure by interacting with the PP1 RVxF domain. We then assessed both the potential cytotoxic and immunogenic activity of SIX2G on in vitro models of multiple myeloma (MM), which is an incurable hematological malignancy characterized by an immunosuppressive milieu. We found that the treatment with SIX2G inhibited cell viability by inducing G2/M phase cell cycle arrest and apoptosis. Moreover, we observed the increase of hallmarks of ICD such as CALR exposure, ATP release and phospho-eIF2α protein level. Through co-culture experiments with immune cells, we demonstrated the increase of (i) CD86 maturation marker on dendritic cells, (ii) CD69 activation marker on cytotoxic T cells, and (iii) phagocytosis of tumor cells following treatment with SIX2G, confirming the onset of an immunogenic cascade. In conclusion, our findings provide a framework for further development of SIX2G as a new potential anti-MM agent.

## 1. Introduction

Microtubule targeting agents (MTAs) are commonly used for the treatment of cancer patients within conventional regimens, especially for tumors that lack actionable mutations and/or are resistant to personalized treatments [1,2,3]. These agents interfere with the microtubule (MT) dynamics killing proliferating cells via mitotic arrest and apoptosis. MTAs are conventionally divided into two groups depending on their ability to stabilize or destabilize MT polymers through the binding of one of the seven sites of tubulin. The MT-stabilizing agents (MSAs) are able to bind taxane or laulimalide/peloruside sites, while the MT-destabilizing agents (MDAs) are able to bind vinca or maytansine domain, colchicine, pironectin or gatorbulin sites [2]. However, the classical view of MTAs was overcome over time thanks to the additional molecular mechanisms that emerged as being responsible for their therapeutic effect [4]. Among these mechanisms are the decrease of angiogenesis [5,6] and cell migration [7,8], as well as the stimulation of the immune response [9,10,11]. This evidence rekindled the interest of medical research toward the design of novel MTAs with lower toxicity and increased selectivity to reduce the off-target effects that constitute the major limit for clinical practice [12]. 

In the drug discovery process, molecules binding at the colchicine site attract great attention, as there is no approved candidate so far [13]. Combretastatin A-4 (CA-4) represents a promising lead compound since it has demonstrated potent activity against different cancer cell lines through the inhibition of tubulin polymerization binding at the colchicine site [14,15]. CA-4 can also disrupt tumor vasculature, resulting in necrosis of tumor cells [16]. Cis-restricted CA-4 analogues were synthetized to stabilize the cis configuration of the olefinic double bond, which is crucial for their activity. In particular, 4,5-diarylisoxazoles demonstrated potent antiproliferative activity, ability in the induction of G2/M phase cell cycle arrest and potent antitubulin activity [17,18]. In this context, different classes of tricyclic systems containing the [1,2]oxale ring were synthetized and evaluated for their antitumor activity [19,20,21,22]. Among these classes, [1,2]oxazolo [5,4-e]isoindoles proved to be valuable candidates with GI_50_ at nanomolar level on the National Cancer Institute (NCI) full panel and in experimental cancer models impairing microtubule assembly and inducing G2/M phase cell cycle arrest and caspase- dependent apoptosis [20,21,23].

Multiple Myeloma (MM) is a plasma cell malignancy characterized by the accumulation of tumor cells in the bone marrow (BM) by the secretion of a monoclonal immunoglobulin also named M-protein and by multi-organ damage [24]. The high genetic heterogeneity and the crucial role of the BM milieu (BMM) remarkably limit the efficacy of available therapeutics [25,26,27,28]. The introduction of immunomodulatory drugs, such as thalidomide or lenalidomide, and proteasome inhibitors such as bortezomib and carfilzomib, has improved patients’ clinical outcome [24,29]. Nevertheless, almost all MM patients relapse and/or become refractory to current therapies [30]. The development of resistant clones mostly depends on BMM cell types that sustain immune-escape and drug resistance by leading to patients’ death. To overcome these hostile conditions, novel therapeutic strategies should be designed with a multi-targeting purpose by combining cytotoxic drugs with agents modulating the BMM, including its immunogenic activity. Notably, several cytotoxic drugs, including oxaliplatin, cyclophosphamide, bortezomib, idarubicin, mitoxantrone, epirubicin, and doxorubicin, have been recently described as inducers of Immunogenic Cell Death (ICD) [31].

ICD has been defined as “*a regulated cell death that is sufficient to activate an adaptive immune response in immunocompetent syngeneic hosts*”, according to the Nomenclature Committee on Cell Death [32]. This complex mechanism is able to trigger a tumor-targeting immune response due to the increased antigenicity of tumor cells and their ability to produce adjuvant signals [33]. The main hallmarks of ICD include the phosphorylation of eIF2α, the extracellular secretion of ATP, and, most importantly, the cell-surface translocation of Calreticulin (CALR) [34,35]. One of the current hypotheses to explain CALR exposure leading to the induction of ICD is the disruption of the PP1/GADD34 complex that composes eIF2α phosphatase [36]. This event, together with the exposure at the other so-called damage-associated molecular patterns (DAMPs) in the tumor microenvironment (TME), stimulates the antitumor immune system by driving immature dendritic cells (DCs) toward a mature phenotype able to promote the tumor-antigen processing machinery and then to activate T-cell mediated killing and phagocytosis. Moreover, the induction of ICD contributes to long-lasting protective anticancer immunity [37]. 

In the era of immunotherapeutic approaches [38,39,40], the improvement of immune response by using MTAs should be considered of great relevance for treatment of cancers characterized by an immunosuppressive TME. In this regard, we endeavored to assess the anti-tumor activity of the novel MTA SIX2G, a derivative of a new class of tricyclic compounds containing the [1,2]oxazole ring and an isoindole moiety, on in vitro models of MM. Together with the cytotoxic activity, we investigated its role as an ICD inducer, which has to this point been uncharacterized for this specific class of drugs. Our findings highlight the potential implication of an immunogenic MTA for the treatment of MM as well as for other malignancies that could take advantage of this therapeutic approach, including relapsed/refractory solid tumors.

## 2. Results

### 2.1. SIX2G Could Sterically Prevent PP1/GADD34 Association by Inducing Immunogenic Cell Death

**Figure 1:**
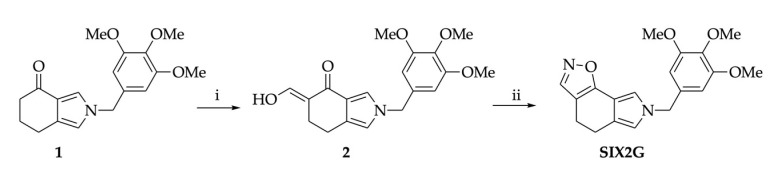
**Reagents and conditions for SIX2G chemical synthesis.** (**i**) t-BuOK, toluene, 0 °C to RT, 2 h then HCOOEt, RT, 24 h, 64%; (**ii**) NH2OH·HCl, ethanol, reflux, 50 min, 61%.

SIX2G was synthetized starting from tetrahydroisoindolone 1 following the synthetic pathway reported in Figure 1. Ketone 1, prepared as we previously reported [21], was converted in the key intemediate 2 by introduction of a hydroxymethylene functionality in α position using ethyl formate as formylating agent and potassium t-butoxide, as base, in toluene (64%) (Figure 1i). The latter was reacted with hydroxylamine hydrochloride, as dinucleophile, in ethanol to yield SIX2G (61%) (Figure 1ii). 

We observed a chemical similarity between SIX2G and some active compounds able to target the RVxF-binding cavity of the PP1 subunit (Figure S1 [41,42,43]). Specifically, these compounds are characterized by an aromatic tricyclic core and by a methoxysubstituted N-benzyl moiety, which we also find in the SIX2G structure. In this regard, we applied computational techniques to investigate the potential activity of SIX2G to induce CALR exposure by means of the dissociation of PP1/GADD34 complex, which constitutes eIF2α phosphatase. Thus, in the docking simulations, we focused our attention on the RVxF PP1 domain and observed that SIX2G exhibited G-score values comparable to that of known active compounds in all 3D models of the RVxF domain (Table S1). Indeed, G-score values of SIX2G fall within the average cut-off of known active compounds for both chains of 4XPN and 3EGG PDB models and chain A of 3E7A. However, for the other 3D models considered, the G-Score values of our compound are very close to the average value, always showing a difference of less than 0.5 kcal/mol. We then conducted a thermodynamic analysis on SIX2G complexes to further strengthen our computational data (Table 1). The complexes of SIX2G with PP1 reached good ΔG_bind_ values, ranging between −39.70 and −57.33 kcal/mol, supporting the ability of our compound to advantageously bind the RxVF domain. By investigating the single contributions of the ΔG_bind_ energy components, we observed the hydrophobic term (ΔG_Lipo_ and ΔG_vdW_) as the driven force in the binding process towards the RVxF domain.

**Figure 2:**
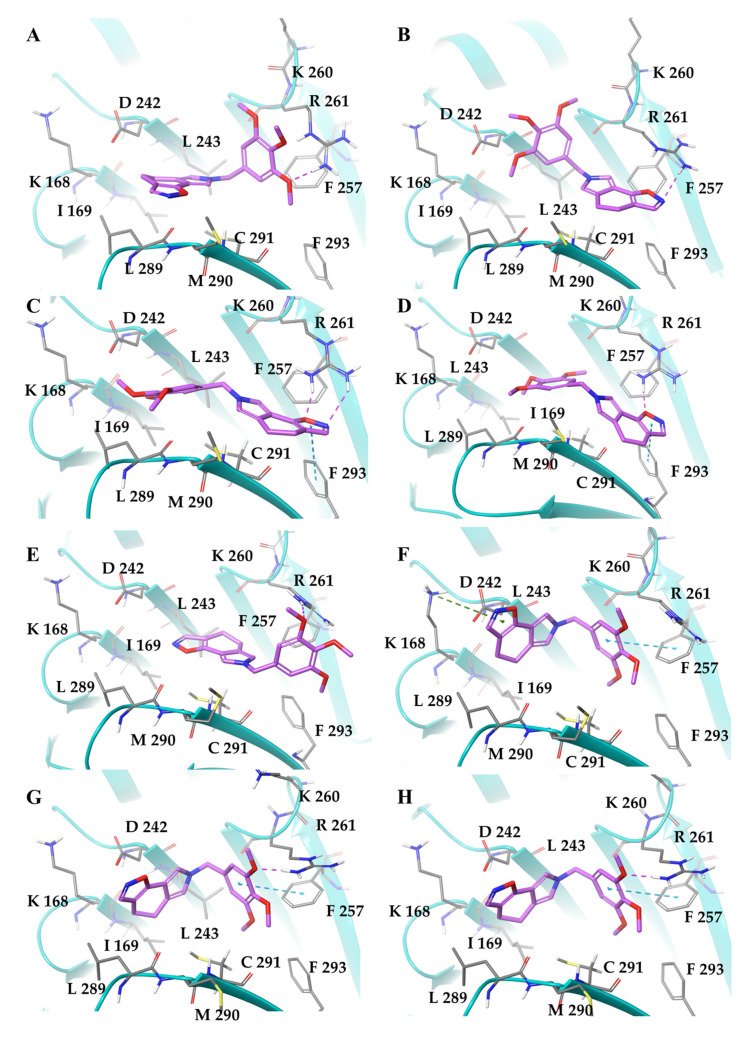
**3D representation of the best-docked poses of SIX2G with the RVxF domain.** PDB models: (**A**,**B**) 3E7A, (**C**,**D**) 3E7B, (**E**,**F**) 4XPN and (**G**,**H**) 3EGG. For all the 3D structures, both chains A (subfigures (**A**,**C**,**E**,**G**)) and B (subfigures (**B**,**D**,**F**,**H**)) were considered and analyzed. PP1 is shown in teal cartoons, while the ligand and the most important residues, involved in the binding event, are shown as magenta and grey thick tube, respectively. In the 3D representation, H-bonds, π-cation and stacking interactions are indicated as dashed violet, green and cyan lines, respectively.

Finally, we visually analyzed the best binding poses obtained by docking simulations for all 3D models of PP1 (Figure 2). Interestingly, we observed that all binding poses with the best ΔG_bind_ values shared the same orientation in the RVxF domain, characterized by the 3,4,5-trimethoxybenzyl substituent facing the residues R-261 and F-257, allowing it to establish an H-bond and a π-π interaction, respectively (Figure 2A,E–H). On the other hand, the tricyclic moiety is placed in a hydrophobic pocket made up of the residues I169, L243 and L289, forming several good contacts mirroring the most favorable lipophilic and van der Waals components of ΔG_bind_. By examining the other docking poses, we pointed out an upside-down binding mode, characterized by a worse binding free energy (Figure 2B–D). Here, the tricyclic portion is directed towards the R-261 and F-257 by establishing one or two H-bonds and a π-π interactions with the side chains of the aforementioned residues, respectively.

In the light of collected computational data, it seems plausible to claim that SIX2G can be considered a binder of the RVxF domain by acting as an ICD inducer. Therefore, our in silico prediction suggests to evaluate not only the cytotoxic, but also the immunogenic activity of this molecule. 

### 2.2. SIX2G Inhibits Cell Viability and Induces Apoptosis of Multiple Myeloma Cells 

**Figure 3:**
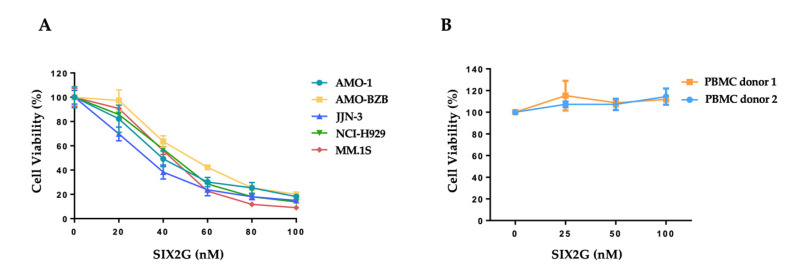
**Dose-response curves.** CellTiter-Glo^®^ Luminescent Cell Viability assay was conducted on tumoral MM cell lines (**A**) and on PBMC from healthy donors (**B**) 48 h after treatment with SIX2G at the indicated concentrations. Dose-response curves represent the percentage (%) of cell viability ± standard deviation (SD) of treated cells. Absorbance values were normalized on values from DMSO treated cells (vehicle) to calculate the represented percentage.

The cytotoxic effect of SIX2G was evaluated on a panel of 5 MM cell lines that are representative of the major genomics and cytogenetics aberrations of MM patients. Among the cell lines tested, we included AMO-1 and AMO-1 bortezomib resistant cells (AMO-BZB) to evaluate the effect of SIX2G on an in vitro model reflecting the acquired resistance to this approved anti-MM agent. MM cells were treated with increasing concentrations of SIX2G (from 20 nM to 100 nM) and then cell viability and apoptosis were evaluated. We observed a dose-dependent (Figure 3A) and time-dependent (Figure S2) inhibition of cell viability on all the MM cell lines tested, with EC_50s_ ranging from 40 to 60 nM (Figure 3A). We then evaluated the potential cytotoxic effect of SIX2G on non-tumoral cells by exposing PBMCs from healthy donors, for 48 h, to increasing doses of the molecule. No effect on cell viability was observed on treated PBMCs, suggesting a favorable therapeutic index (Figure 3B). 

**Figure 4:**
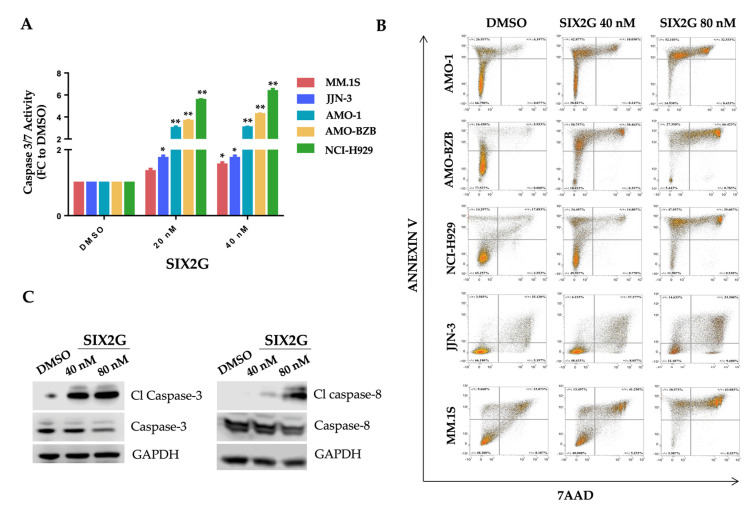
**Apoptosis Assays.** The activation of caspases-3 and caspase-7 was evaluated through the Caspase Glo^®^ assay. Histogram bars represent mean ± SD of the fold changes (FC) of absorbance values of SIX2G treated cells respect than cells treated with DMSO (**A**). Dot plots in figure (**B**) represent the distribution of cells treated for 24 h with DMSO or SIX2G at the indicated concentrations, following Annexin V/7AAD staining (**B**). Representative WB analysis of caspase-3, caspase-8, cleaved (Cl) caspase 3 and Cl-caspase 8 detected on AMO-1 cells 24 h after SIX2G treatment at the indicated concentrations. GAPDH was used as loading control (**C**) (Whole WB images and densitometry analyses are reported in Supplementary Information). Student’s *t*-test was applied to calculate the statistical significance between multiple group comparisons, respect than DMSO treated cells. *p*-value < 0.05 is indicated with one star (*), *p*-value < 0.01 with two stars (**).

Moreover, through the Caspase-Glo assay, we observed an increase of caspase 3/7 activity that is indicative of the onset of apoptotic pathways at sub lethal doses and exposure-time (24 h) (Figure 4A). Flow cytometric analysis of Annexin V/7AAD stained cells, confirmed the dose-dependent induction of apoptosis within 24 h of drug exposure at 40 nM and 80 nM, respectively (Figure 4B). A representative WB analysis of cleaved caspase-3 and cleaved caspase-8 protein levels, conducted on AMO-1 cells, is showed in Figure 4C and demonstrated the activation of both extrinsic and intrinsic apoptotic pathways.

### 2.3. SIX2G Induces Alteration in Mitotic-Spindle and Apical Junction Pathways and Triggers Cell Cycle Arrest in G2/M Phases 

**Figure 5:**
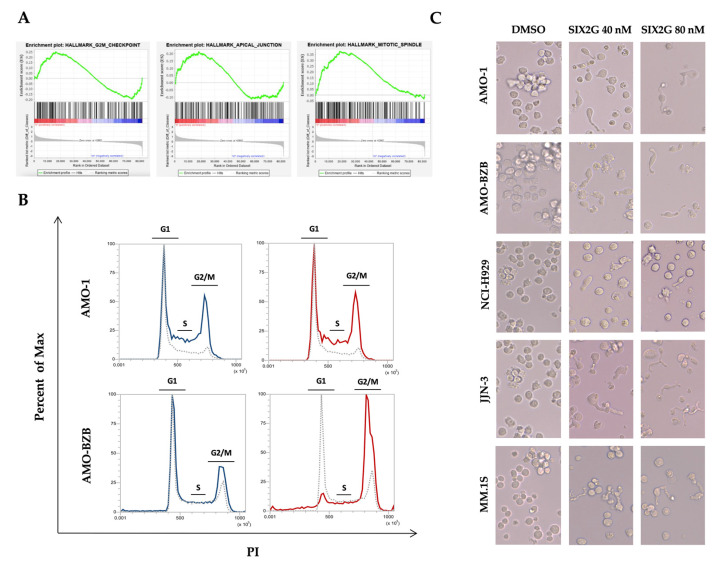
**Evaluation of MTAs-induced effects.** GSEA was performed on data obtained from GEP of AMO-1 cells treated with SIX2G at the EC_50_ concentration (40 nM). Differentially expressed genes (SIX2G vs DMSO treated cells) were identified by applying a FC ≤ −1.5 or ≥1.5 and a *p*-value < 0.05. The mainly perturbed pathways are represented (**A**). Representative flow cytometric analyses of PI stained cells are indicative of cell cycle distribution of AMO-1 and AMO-BZB 24 h after treatment with SIX2G at 40 nM (Blue Line) and 80 nM (Red line). Data were compared with respect to DMSO treated cells (gray dashed line) (**B**). Representative pictures of the morphological changes induced by SIX2G 24 h after treatment (**C**) Magnification 40X.

We performed gene expression profiling (GEP) on AMO-1 cells after 48 h of exposure to SIX2G at EC_50_ concentration in order to highlight the transcriptional changes following SIX2G treatment. According to the effects induced by MTAs, the GSEA revealed the modulation of pathways underlying a cell cycle deregulation as well as apical junction and mitotic-spindle pathways (Figure 5A). The effect of these changes, probably triggered as a result of a short exposure time, is evident on cells as early as 24 h after treatment. As matter of fact, cell cycle profiling confirmed the expected accumulation of cells in G2/M, 24 h after treatment (Figure 5B). At the same point in time, a morphological evaluation of SIX2G treated cells confirmed the cellular elongation typically observed after MTAs exposure (Figure 5C). 

### 2.4. SIX2G Induces Calreticulin Exposure and ATP Release by Activating an Immunogenic Cascade 

**Figure 6:**
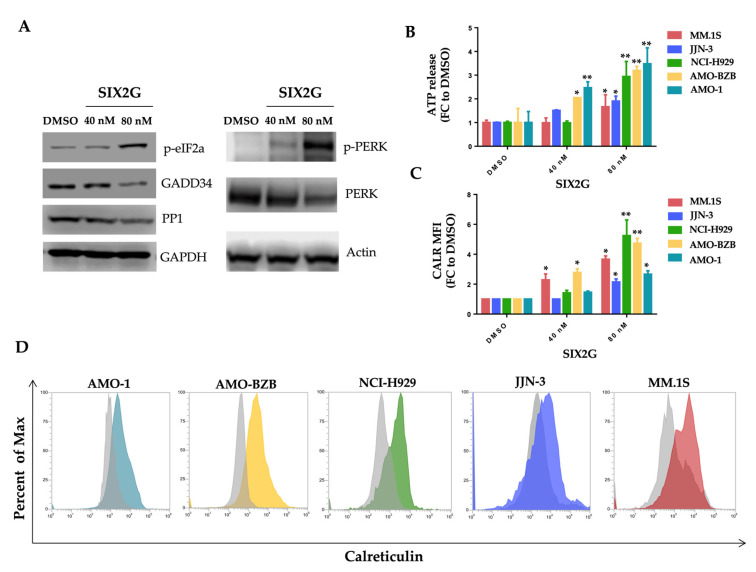
**Assessment of the onset of ICD.** Representative WB analysis of PP1, GADD34, phosphorylated-eIF2α (p-eIF2α), PERK and phosphorylated-PERK (p-PERK) conducted on AMO-1 cells, 48 h after treatment with SIX2G. GAPDH or Actin were used as loading controls (**A**). The release of ATP in cell supernatant was detected through a Cell Titer Glo^®^ Assay. Histogram bars represent mean ± SD of the FC of absorbance values from cells treated with SIX2G respect to cells treated with DMSO (**B**). CALR exposure to the cell membrane was evaluated through flow cytometry. Histogram bars represent mean ± SD of FCs of median fluorescence intensity (MFI) values of CALR signal in cells treated with SIX2G respect to DMSO (**C**). Representative images of flow cytometric analyses of CALR membrane exposure 48 h after treatment with SIX2G at 80 nM (colored curves) or DMSO (gray curves) are reported (**D**). Student’s *t*-test was applied to calculate the statistical significance between multiple group comparisons, respect than DMSO treated cells. *p*-value < 0.05 is indicated with one star (*), *p*-value < 0.01 with two stars (**).

Since computational studies suggested a molecular mechanism enabling SIX2G to activate an immunogenic cascade, we analyzed the main hallmarks of this pathway. We firstly assessed the increase of eIF2α phosphorylation that is considered pathognomonic for ICD [44]. WB analysis highlighted a decrease in both PP1 and GADD34 subunits of eIF2α-phosphatase and the expected increase of phosphorylated-eIF2α in a dose dependent manner (Figure 6A right panel). Moreover, we observed an increase in the phosphorylation level of the kinase PERK that is adjuvant for eIF2α phosphorylation (Figure 6A left panel).

We then evaluated the release of ATP in the cell culture supernatant and the membrane translocation of CALR on SIX2G treated cells. We highlighted a dose dependent increase in the ATP release from MM cells within 48 h of treatment with the molecule (Figure 6B). Moreover, 48 h after treatment with SIX2G at 40 nM and 80 nM, we observed a significant increase of CALR exposure on the membrane of MM cell lines (Figure 6C,D) by indicating the onset of ICD. Importantly, cells have been stained with 7AAD to exclude apoptotic cells from the evaluation of CALR exposure. Altogether, this data support our hypothesis.

### 2.5. SIX2G Treatment on Tumor Cells Promotes DC Maturation, Activation and Cross-Priming

**Figure 7:**
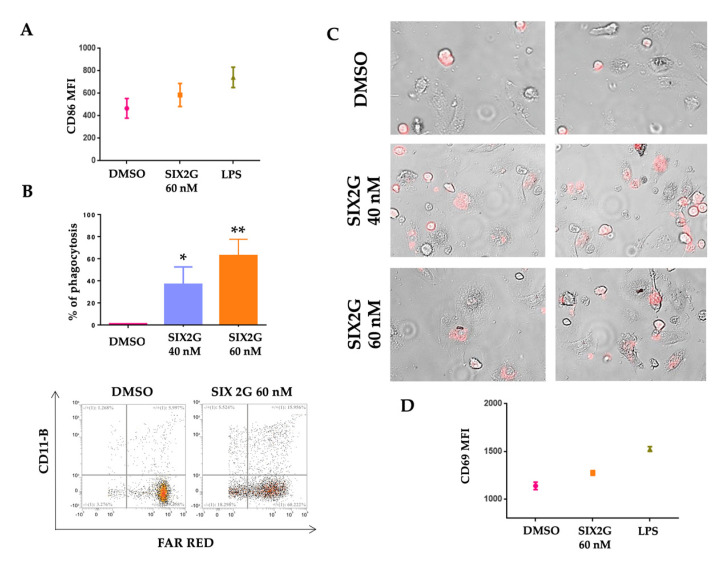
**Assessment of DC maturation and phagocytosis.** Representative figure of flow cytometric analyses conducted on DMSO or SIX2G pre-treated AMO-1 cells after 24 h of co-culture with DCs (E:T of 5:1). Scatter plots represent mean ± SD of MFI of CD86 signal, stimulation with lipopolysaccharide (LPS) was used as positive control of DC activation (**A**). Histogram bars represent the % of phagocytosis of cells treated with SIX2G with respect to DMSO ((**B**) **upper** panel). This percentage was calculated as follows: mixed AMO-1 Far Red^+^ cells and DCs were stained with CD11b, and with 7AAD to exclude apoptotic cells. Phagocytosed AMO-1 cells were detected as CD11b^+^, FAR RED^+^ and 7AAD^−^ cells. (Representative dot plots of flow cytometric analysis are represented in figure (**B**) **lower** panel). Representative figures of immunofluorescence assay show the engulfment by DCs of Far Red stained AMO-1 cells. DMSO treated AMO-1 cells appear round and in suspension respect than DCs that are instead attached to the bottom of the plate, AMO-1 cells treated with SIX2G at 40 nM and 60 nM appear as engulfed in DCs (**C**) Magnification 40X, scatter plots in figure (**D**) represent mean ± SD of MFI of CD69 CTLs activation marker. CD69 signal was detected on CTLs after 24 h of co-culture with AMO-1 cells pre-treated with SIX2G at 60 nM (DMSO and LPS were used as negative and positive controls, respectively). Student’s *t*-test was applied to calculate the statistical significance between multiple group comparisons, respect than DMSO treated cells. *p*-value < 0.05 is indicated with one star (*), *p*-value < 0.01 with two stars (**).

ICD dying cancer cells are able to induce antigen presenting cell (APC) maturation and to improve the ability to be engulfed by APC through phagocytosis. To investigate if SIX2G promotes DC maturation and activation, we pre-treated AMO-1 cells with DMSO or SIX2G for 48 h. For co-culture assays, we selected 60 nM as the highest concentration of SIX2G that allowed us to perform a clear evaluation of the onset of ICD on a significant percentage of surviving cells. We co-cultured human healthy donor derived DCs with SIX2G pre-treated AMO-1 cells at a 5:1 ratio. After 24 h of co-culture we assessed DC activation markers via a flow cytometry-based assay. Interestingly, AMO-1 pre-treated with SIX2G at 60 nM are able to increase the expression of the CD86 activation marker on the surface of co-cultured DCs (Figure 7A) as compared to AMO-1 DMSO pre-treated cells. Next, we investigated if SIX2G could improve the ability of co-cultured DCs to engulf MM dying cancer cells. For this purpose, we labelled DMSO and SIX2G pre-treated AMO-1 cells with Cell Trace Far Red and co-cultured MM cells with DCs at a 5:1 E:T ratio. After 12 h of co-culture, we collected and stained cells with CD11b, which is a macrophage marker, and we assessed phagocytosis through flow cytometry (Figure 7B) and immunofluorescence assays (Figure 7C). Importantly, we observed that SIX2G pre-treated AMO-1 cells showed a significantly increased engulfment by DCs in a dose dependent manner as compared to DMSO pre-treated AMO-1 (Figure 7B upper panel). Phagocytosed AMO-1 cells were detected as positive for CD11b and Far Red and negative for 7AAD to exclude apoptotic cells from the analysis (Figure 7B lower panel). 

To investigate if DCs exposed to MM cells undergoing ICD are able to activate cytotoxic lymphocytes (CTLs), we co-cultured AMO-1 cells pre-treated with DMSO or SIX2G at 60 nM with healthy donor-derived DCs and autologous CTLs for 24 h. Finally, we demonstrated that pre-treated SIX2G cells are able to induce T cell activation as shown by the increased surface expression of CD69 (Figure 7D) as compared to DMSO pre-treated cells.

## 3. Discussion

ICD is a unique form of regulated cell death characterized by the active or passive release of several DAMPs including CALR and the small metabolite ATP [45]. DAMPs are recognized by pattern recognition receptors (PRRs) expressed on innate and adaptive immune system cells resulting in DC activation, maturation and cross-presentation of antigens to CTLs [35]. This cascade of events can drive a robust immune activation that leads to a radical skewing from “cold” to “hot” immune TME. Several anti-cancer agents such as conventional chemotherapeutics (cyclophosphamide, platinum derivatives, anthracyclines), tyrosine kinase inhibitors (crizotinib, cabozantinib), proteasome inhibitors (bortezomib) and radiotherapy, recently emerged as potent ICD inducers. Moreover, different cellular stressor agents could induce ICD, among them, are to be mentioned the MTAs such as taxanes, auristatin F and colchicine [33]. 

MM is a hematological malignancy that arises from uncontrolled abnormal plasma cell proliferation within BM. Over the past decades, the survival rate of MM patients has been radically improved by the introduction of new therapeutic regimens. However, despite the huge improvement in the treatment, MM remains an incurable disease due to the emergence of drug resistance and recurrent relapses [46]. Although genetic alterations have a certain role in MM pathogenesis, many of them are already present in the pre-malignant stage, suggesting that genetic mutations are necessary but not sufficient for MM progression [47]. Other extrinsic factors such as the interaction with immunosuppressive TME could be involved in MM evolution. In this regard, there is strong evidence for a progressive impairment in both innate and adaptive immune response from pre-malignant condition to malignant MM disease.

MM TME is characterized by the presence of several immunosuppressive cellular and non-cellular elements including myeloid-derived suppressor cells (MDSCs), tumor-associated M2-like macrophages, regulatory T-cells, and tumor promoting and immunomodulatory factors such as IL-6, TGF-β, IL-10, PGE2 [48]. The complex interplay between MM cells and TME components results in immune dysfunction and finally in MM immune escape. On these premises, new therapeutic strategies directed to revert immunosuppression and to increase immunogenicity could be effective even in relapsed/refractory and heavily pre-treated MM patients.

Here we report the development, preclinical validation and characterization of SIX2G, a novel small molecule belonging to the MTA class. We provided a computational model able to predict the molecular mechanisms that may underlie the triggering of ICD and that could be applied to other ICD-inducers whose mechanism of action remain unclear. Through our in-silico prediction, we hypothesized that SIX2G could sterically interfere with the PP1/GADD34 complex by inducing ICD. Next, we demonstrated for the first time that SIX2G promotes cell cycle arrest and apoptosis on in vitro MM models. We demonstrated that SIX2G acts as ICD inducer on MM cells as showed by increased CALR exposure, extracellular ATP release and p-eIF2α protein level. Finally, we demonstrated that SIX2G could elicit MM immunogenicity and effectively promote immune function as suggested by the increase in maturation of DC co-cultured with MM cells, promotion of cancer cell phagocytosis and CTL activation. Interestingly, our data highlighted the ability of SIX2G to act as cytotoxic MTA and as ICD inducer on a cellular model represented by the AMO-BZB cell line, which reflects the acquired resistance to bortezomib that commonly occurs in MM patients. Recently, the proteasome inhibitor bortezomib has emerged as an ICD trigger in MM by inducing a two-to-three-fold CALR exposure increase on the surface of the malignant PCs and by improving the anti-tumor activity of the DCs [49,50]. Our data demonstrated a similar fold increase (from two to five) after SIX2G treatment on MM cell lines, including bortezomib resistant cells.

In conclusion, our findings highlighted the ability of SIX2G to exert a significant and selective anti-MM activity in vitro. Moreover, our results supported the molecular rationale for the induction of the ICD. The relevance of these data is to be considered in the context of the treatment of orphan diseases in which the immunosuppressive microenvironment impairs the effectiveness of presently available treatments. Future studies will be performed on in vivo models to determine the therapeutic dose of SIX2G for MM patients, as well as to perform pharmacokinetic and pharmacodynamics assessments in the perspective view to move our results from bench to bedside. 

## 4. Materials and Methods

### 4.1. Chemical Synthesis of SIX2G 

Chemical synthesis of 7-(3,4,5-trimethoxybenzyl)-5,7-dihydro-4H-[1,2]oxazolo [5,4-e] isoindole (SIX2G) was performed starting from tetrahydroisoindolone 1 by subsequent chemical modifications. A detailed description of methods used for chemical synthesis is provided in Supplementary Material and Methods [21].

### 4.2. Docking Simulations

Docking studies and thermodynamics analysis were performed with the aim of investigating the interaction between SIX2G and the RVxF domain of serine/threonine protein phosphatase 1 (PP1). In particular, the Schrödinger Suite version 2018 was employed as a computational tool for carrying out all molecular modeling simulations [Schrödinger LLC, New York, NY, USA, (2018)], while different crystal structures of PP1 protein were downloaded from the Protein Data Bank (PDB) [51].The protocol details were reported in the Supplementary Material and Methods [51,52,53,54,55,56,57,58,59,60,61].

### 4.3. Cell Cultures

MM cell lines NCI-H929, MM.1S, and JJN-3 were purchased from DSMZ, while AMO-1 and AMO-BZB cells were kindly provided by Dr. C. Driessen (University of Tubingen, Tubingen, Germany). All cellular models have been tested for mycoplasma before use. Whole peripheral blood was obtained from healthy blood donors who provided informed consent according to our institutional bioethical requirement. Peripheral blood mononuclear cells (PBMCs) were isolated from each buffy coat by density gradient centrifugation using Lympholyte Cell Separation Media (Euroclone, Milan, Italy). The culture media was RPMI-1640 supplemented with 10% FBS, 2 μmol/L glutamine, 100 U/mL penicillin, and 100 μg/mL streptomycin (GIBCO; Thermo Fisher Scientific, Waltham, MA, USA). 

### 4.4. Dentric Cells Generation

DCs were generated from healthy donor derived PBMC by using Ficoll-Paque Plus Lympholyte density gradient centrifugation. CD14^+^ monocytes isolation was performed through immunomagnetic cell sorting using CD14 microbeads (MACS Miltenyi Biotec, Bergisch Gladbach, Germany) according to the manufacturer instructions. CD14^+^ cells were seeded in RPMI-1640 medium containing GM-CSF and IL-4 (130-094-812; Miltenyi Biotec, Bergisch Gladbach, Germany) in 5% CO_2_ at 37 °C.

### 4.5. Cell Viability, Apoptosis and Cell Cycle Analysis

Cell viability and Caspase Activity assays have been performed through CellTiter Glo and Caspase-Glo 3/7 assays (Promega, Madison, WI, USA), respectively, according to manufacturer instructions, within 24 h of treatment with DMSO (vehicle) or SIX2G; the luminescence was recorded by using a Glomax multi-detection system (Promega, Madison, WI, USA).

Apoptosis was evaluated 24 h after treatment by flow cytometry following Annexin V/7AAD staining (BD, Franklin Lakes, NJ, USA).

Cell cycle profiling of DMSO or SIX2G treated cells was evaluated within 24 h of treatment by flow cytometry following Propidium Iodide (PI) staining. In this regard, cells were collected, washed in phosphate-buffered saline (PBS) and fixed in ice-cold 70% ethanol overnight at −20 °C. Fixed cells have then been stained with 50 μg/mL PI, 100 μg/mL RNase, 0.05% Nonidet P-40 (NP40) for 1 h in the dark at room temperature. Cell cycle distribution was then detected through Attune NxT Flow Cytometer (Thermo Fisher Scientific, Waltham, MA, USA). 

### 4.6. Gene Expression Profiling 

AMO-1 cells were exposed to DMSO or SIX2G 40 nM for 48 h. All treatments were performed in biological duplicate. Cells were harvested and used for total RNA extraction by using the RNeasy Mini kit (Qiagen, Hilden, Germany). For each sample, 100 ng total RNA was processed by the GeneChip^®^ WT PLUS Reagent Kit (Thermo Fisher Scientific, Waltham, MA, USA) according to the manufacturer’s instructions and then hybridized to the GeneChip^®^ Clariom D Human Array (Thermo Fisher Scientific, Waltham, MA, USA). Hybridization, wash, stain, and scan were carried out with a GeneChipTM 3000 system. Probe intensity files (CEL files) were generated by the GeneChip^®^ Command Console software and analyzed using the Transcriptome Analysis Console software v.4.0 (Applied Biosystem by Thermo Fisher Scientific, Waltham, MA, USA). The Signal Space Transformation-Robust Multi-Array Average (SST-RMA) algorithm was applied to array data background adjustment, normalization, summarization, and log-transformation of signals intensity. Differentially expressed genes (SIX2G versus DMSO) were identified by applying a fold change (FC) ≤ −1.5 or ≥1.5 and a *p*-value < 0.05. Annotation of data was also performed using the Clariom D human library available on TAC 4.0. The gene lists were applied to gene set enrichment analysis (GSEA) software to reveal the most significant biological pathways modulated by SIX2G using a false discovery rate ≤ 0.25 in 1000 permutations.

### 4.7. Western Blot Analysis 

Whole protein extracts were recovered from cells after treatment with DMSO or SIX2G by using NP40 lysis buffer complemented with Halt Protease and Phosphatase Inhibitor cocktail (Invitrogen by Thermo Fisher Scientific, Waltham, MA, USA). Western blot (WB) analysis was performed as previously reported [26]. The primary antibodies used were: PERK (#3192), eIF2α (#5324), p-eIF2α (Ser 51) (#3398), Caspase-3 (#9662), Cleaved Caspase-3 (Asp 175) (#9661), Caspase-8 (#4790) and Cleaved Caspase-8 (#9748) by Cell Signaling Technology (Danvers, MA, USA); p-PERK (Thr 981) (#orb336657), b-actin (#Sc-1616), GAPDH (SC-25778), GADD34 (SC-46661) and PP1 (SC-7482) by Santa-Cruz (Dallas, TX, USA). Anti-rabbit IgG HRP-linked antibody (#7074, Cell Signaling Technology, Danvers, MA, USA), anti-mouse IgG HRP-linked antibody (#7076, Cell Signaling Technology, Danvers, MA, USA) and anti-goat IgG HRP-linked antibody (SC-2354, Santa Cruz, Dallas, TX, USA) were used as secondary antibodies depending on the host species of animal in which the primary antibodies were raised. 

### 4.8. Calreticulin Exposure and ATP Release Analyses 

Indirect staining for flow cytometry analysis was performed as previously described [62]; briefly: 200.000 cells were collected 24 h after treatment with DMSO or SIX2G and centrifuged at 1200 rpm for 5 min (these setting were maintained for all the centrifugation steps). After washing, each cell pellet was incubated for 20 min at room temperature in the dark with 1 µg/mL of isotype control (™) or 1 µg/mL of anti-Calreticulin antibody (ab2907 Abcam). After that, cells were rinsed in 2 mL of washing buffer and then stained with 1 µL of Alexa-fluor 488 goat anti-rabbit IgG H + L (#A11008, Invitrogen, Thermo Fisher Scientific) in a final volume of 100 µL of washing buffer. After 20 min of incubation at room temperature protected from light, samples were washed and resuspended in 500 µL of PBS for flow cytometry analysis. Signals were then acquired via Attune NxT Flow Cytometer (Thermo Fisher Scientific, Waltham, MA, USA). 

For the evaluation of ATP release, cell supernatants were collected 24 h after treatment with DMSO or SIX2G and centrifuged at 12,000 rpm at 4 °C for 6 min. An ATP analysis was then immediately performed by using a CellTiter-Glo^®^ Luminescent Cell Viability Assay kit (Promega, Madison, WI, USA) as described by the manufacturer. The luminescence was recorded by using the Glomax multi-detection system (Promega, Madison, WI, USA). 

### 4.9. DC Maturation and Phagocytosis Assay 

Immature DCs were cultured alone or in co-culture with AMO-1 pre-treated for 48 h in the presence of lipopolysaccharide (LPS) or DMSO or SIX2G 60 nM, at a 5:1 Effector: Target (E:T) ratio. After 24 h of co-culture, cells were collected, washed in PBS and then stained with anti–CD86-PeCy5 (#555666, BD, Franklin Lakes, NJ, USA), and 7AAD (#51-68981E, BD, Franklin Lakes, NJ, USA). Dead cells were excluded by 7AAD positivity, and CD86 DC maturation marker expression was evaluated by flow cytometry. 

For phagocytosis assay, AMO-1 were labelled with CellTrace Far Red (Thermo Fisher Scientific, Waltham, MA, USA), treated with DMSO or SIX2G 60 nM for 48 h, and then co-cultured for 12 h at a 5:1 ratio with monocyte-derived DCs (Mo-DCs). Cells were then detached, collected and stained with CD11b-PeCy7 (#557743, BD, Franklin Lakes, NJ, USA) and 7AAD viable marker (BD, Franklin Lakes, NJ, USA) to exclude apoptotic cells. A phagocytosis analysis was performed by flow cytometry. The percentage of phagocytosed cells was evaluated as the percentage of 7AAD^−^ CD11b^+^ Far Red^+^ cells.

### 4.10. DC Cross-Priming 

T cells were selected from CD14^−^ PBMCs from the same donors of DCs using CD3 microbeads (Miltenyi Biotec, Bergisch Gladbach, Germany) and cultured until immature DCs were generated. Either DMSO or SIX2G (60 nM) pre-treated AMO-1 were co-cultured with DCs and T cells for 24 h. T-cell populations were analyzed using the following antibodies purchased by BD (Franklin Lakes, NJ, USA): CD3-PerCPCy5.5 (#560835), CD8-APCH7 (#641400), CD4-FITC (#345768), and CD69-PE (#555531).

### 4.11. Statistical Analysis 

Each experiment was performed in at least three independent biological replicates. Data were reported as mean ± SD. A student’s *t*-test was applied to calculate the statistical significance between multiple group comparisons, respect than cells treated with DMSO. Graphical representations and *p*-Values were obtained by using Graphpad Prism V.6.0. The cut-off to consider a result as statistically significant was fixed on *p* < 0.05. If the *p*-value was <0.05, it was indicated in figures with one star (*), *p*-value < 0.01 with two stars (**), and *p*-value < 0.001 with three stars (***).

## Figures and Tables

**Table 1:** **ΔGbind values and their single contributions.** ΔG_bind_ values are expressed as electrostatic (ΔG_Coul_), lipophilic (ΔG_Lipo_), solvation (ΔG_Solv_) and van der Waals (ΔG_vdW_) components, calculated for all best complexes of SIX2G with the different 3D structures of the protein PP1, obtained by means of docking simulations. All ΔG values are expressed as kcal/mol.

PDB Model	ΔG_bind_	ΔG_Coul_	ΔG_Lipo_	ΔG_Solv_	ΔG_vdW_
3E7A chain A	−51.72	−17.78	−29.04	27.20	−29.61
3E7A chain B	−46.90	−13.12	−25.99	23.24	−30.87
3E7B chain A	−42.50	−7.76	−28.00	22.98	−30.99
3E7B chain B	−39.70	−1.79	−28.62	18.86	−31.61
4XPN chain A	−41.56	−6.96	−22.45	14.25	−22.46
4XPN chain B	−53.17	−13.14	−31.37	23.57	−30.97
3EGG chain A	−57.66	−11.89	−34.15	20.25	−32.64
3EGG chain B	−57.33	−12.70	−33.59	21.36	−30.69

## Data Availability

Not applicable.

## Supplementary Materials

The supporting information can be downloaded at: https://www.mdpi.com/article/10.3390/ijms231810222/s1.
