# Supplementary material for: The New Microtubule-Targeting Agent SIX2G Induces Immunogenic Cell Death in Multiple Myeloma
Source: Int J Mol Sci. 2022 Sep 6;23(18):10222. doi: 10.3390/ijms231810222 (PMC9499408; doi:10.3390/ijms231810222)
Supplement: Supplementary file 1 [file ijms-23-10222-s001.zip › ijms-1875338-SI/Supplementary Figures.pdf]

Supplementary Figure S1

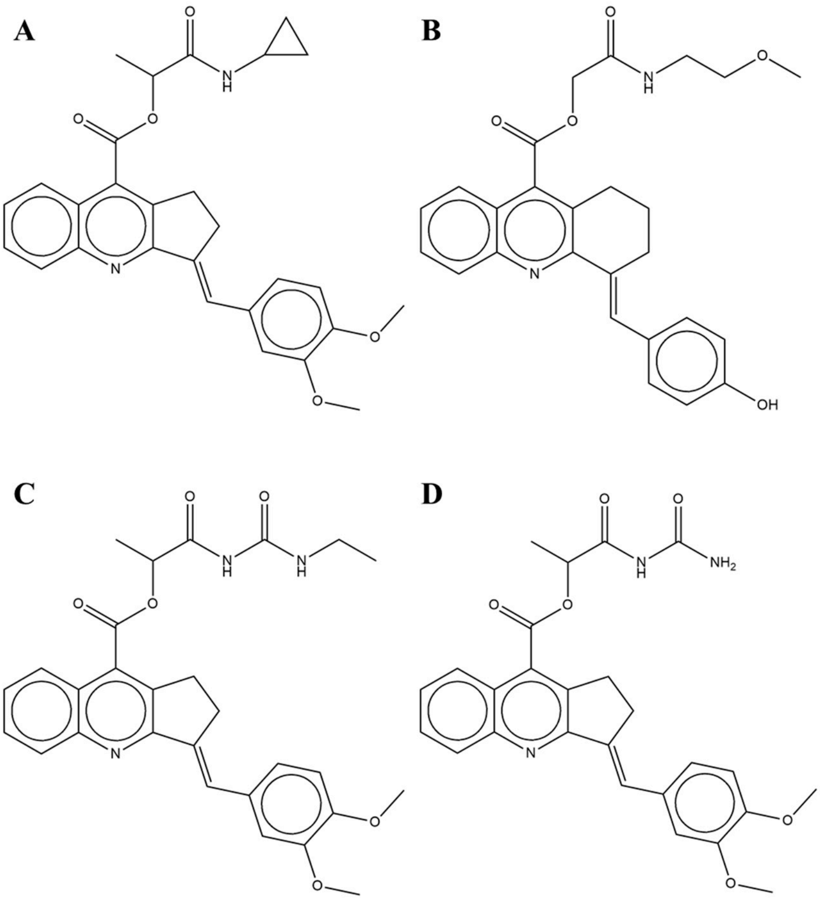

Figure S1. 2D structure of active compounds able to bind RVxF domain of PP1. (A) 1E7-07 (41), (B) 1H4 (42), (C) 1E7-03 (41) and (D) C31 (43), collected from the literature.

References

41. Lin X, Ammosova T, Choy MS, Pietzsch CA, Ivanov A, Ahmad A, et al. Targeting the Non-catalytic RVxF Site of Protein Phosphatase-1 With Small Molecules for Ebola Virus Inhibition. Frontiers in microbiology. 2019;10:2145.

42. Ammosova T, Platonov M, Yedavalli VR, Obukhov Y, Gordeuk VR, Jeang KT, et al. Small molecules targeted to a non-catalytic "RVxF" binding site of protein phosphatase-1 inhibit HIV-1. PloS one. 2012;7(6):e39481.

43. Ammosova T, Pietzsch CA, Saygideger Y, Ilatovsky A, Lin X, Ivanov A, et al. Protein Phosphatase 1-Targeting Small-Molecule C31 Inhibits Ebola Virus Replication. The Journal of infectious diseases. 2018;218(suppl_5):S627-S35.

Supplementary Figure S2

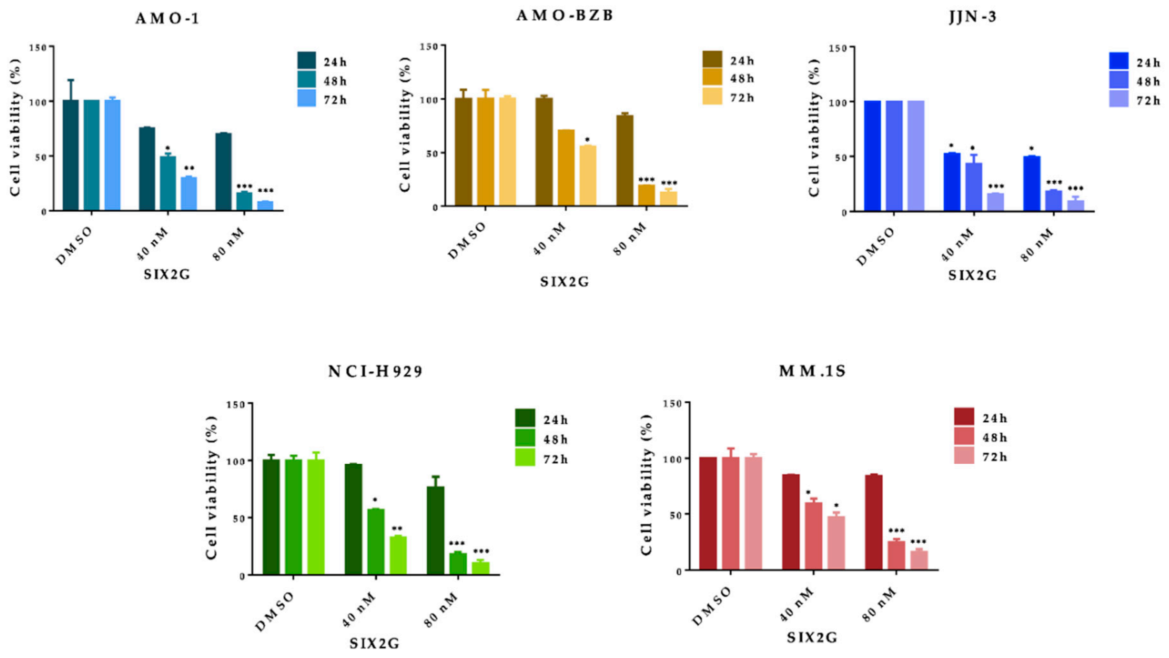

Figure S2. Cell viability assays at different time points. CellTiter-Glo Luminescent Cell Viability assay was conducted on 5 MM cell lines 24 h, 48 h and 72 h after treatment with SIX2G at 40 nM and 80 nM. Histogram bars represent the percentage (%) of cell viability ± standard deviation (SD) of treated cells. Absorbance values of SIX2G treated cells were normalized on values from DMSO treated cells (vehicle) to calculate the represented percentage. Statistical significance was calculated though Stutent’s t-test by comparing each experimental condition (SIX2G doses and time points) respect than the corresponding DMSO one.
