# Supplementary material for: The New Microtubule-Targeting Agent SIX2G Induces Immunogenic Cell Death in Multiple Myeloma
Source: Int J Mol Sci. 2022 Sep 6;23(18):10222. doi: 10.3390/ijms231810222 (PMC9499408; doi:10.3390/ijms231810222)
Supplement: Supplementary file 1 [file ijms-23-10222-s001.zip › ijms-1875338-SI/Supplementary Material and Methods.pdf]

Supplementary Materials and Methods

Chemical synthesis of SIX2G

All melting points were taken on a Bu̇chi melting point M-560 apparatus. IR spectra were determined in bromoform with a Shimadzu FT/IR 8400S spectrophotometer. 1H and 13C NMR spectra were measured at 200 and 50.0 MHz, respectively, in DMSO-d6 or CDCl3 solution using a Bruker Avance II series 200 MHz spectrometer. Column chromatography was performed with Merck silica gel (230–400 mesh ASTM) or a Bu̇chi Sepacor chromatography module (prepacked cartridge system). Elemental analyses (C, H, N) were within ±0.4% of theoretical values and were performed with a VARIO EL III elemental analyzer. The purity of all the tested compounds was >95%, determined by HPLC (Agilent 1100 series).

2-(3,4,5-Trimethoxybenzyl)-2,5,6,7-tetrahydro-4H-isoindol-4-one (1) was prepared according to our published procedure [21].

Synthesis of 5-(hydroxymethylidene)-2-(3,4,5-trimethoxybenzyl)-2,5,6,7-tetrahydro-4H-isoindol-4-one (2)

To a suspension of t-BuOK (3.7 g, 36 mmol) in anhydrous toluene (30 mL), a solution of 1 (12 mmol) in anhydrous toluene (40 mL) was added dropwise under nitrogen at 0 °C. After 3 h stirring at rt the reaction was cooled at 0 °C and a solution of ethyl formate (2.90 mL, 36 mmol) in anhydrous toluene (20 mL) was added and the mixture was kept stirring at room temperature for 24 h, then the solvent was removed at reduced pressure. The residue was dissolved in water and the solution was washed with diethyl ether. The aqueous solution was then acidified with 6 M HCl and the solution was extracted with dichloromethane (3 x 30 mL). The organic phase was dried over Na2SO4 and the solvent evaporated at reduced pressure. The crude product was purified by chromatography column using dichloromethane as eluent. Brown solid; yield 64%; m.p.: 105 - 106 _C; IR cm-1: 2931 (OH), 1631 (CO); 1H NMR (CDCl3, 200 MHz, ppm): 2.49 (t, 2H, J = 7.4 Hz, CH2), 2.65 (t, 2H, J = 7.4 Hz, CH2), 3.83 (s, 6H, 2 x CH3), 3.84 (s, 3H, CH3), 4.96 (s, 2H, CH2), 6.40 (s, 2H, H-20 and H-6’), 6.43 - 6.45 (m, 1H, H-1), 7.27 - 7.29 (m, 1H, H-3), 7.47 - 7.50 (s, 1H, CH), 14.35 (s, 1H, OH); 13C NMR (CDCl3, 50 MHz, ppm): 21.1 (t), 25.9 (t), 54.3 (t), 56.2 (2 _ q), 60.9 (q), 104.7 (2 _ d), 109.4 (s), 117.3 (d), 121.0 (s), 122.5 (d), 125.9 (s), 131.7 (s), 137.8 (s), 153.6 (2 x s), 165.3 (d), 187.0 (s). Anal calcd for C19H21NO5 (343.37): C 66.46, H 6.16, N 4.08. Found: C 66.30, H 5.95, N 4.20.

Synthesis of 7-(3,4,5-trimethoxybenzyl)-5,7-dihydro-4H-[1,2]oxazolo [5,4-e] isoindole (SIX2G)

To a solution of the hydroxymethylideneketone 2 (5.0 mmol) in ethanol (15 mL) hydroxylamine hydrochloride (0.38 g, 5.5 mmol) was added and the reaction mixture was heated under reflux for 50 min. After cooling, the solvent was evaporated at reduced pressure. The crude product was purified by chromatography column using dichloromethane as eluent. White solid; yield 61%; m.p.: 77 - 78 _C; 1H NMR (DMSO-d6, 200 MHz, ppm): 2.65 (s, 4H, 2 x CH2), 3.63 (s, 3H, CH3), 3.76 (s, 6H, 2 x CH3), 4.98 (s, 2H, CH2), 6.71 (s, 2H, H-2′ and H- 6′), 6.81 (s, 1H, H-6), 7.29 (s, 1H, H-8), 8.37 (s, 1H, H-3); 13C NMR (DMSO-d6, 50 MHz, ppm): 19.4 (t), 20.1 (t), 52.7 (t), 55.9 (2 _ q), 59.9 (q), 105.4 (2 _ d), 108.3 (s), 109.9 (s), 115.6 (d), 118.3 (d), 120.0 (s), 133.8 (s), 136.9 (s), 149.1 (d), 152.9 (2 x s), 162.5 (s). Anal calcd for C19H20N2O4 (340.37): C 67.05, H 5.92, N 8.23. Found: C 66.92, H 5.84, N 8.39.

Docking simulations

To evaluate the interaction of SIX2G with the RVxF domain of serine/threonine protein phosphatase 1 (PP1), we performed docking studies by using four different crystal structures of the PP1 protein downloaded from the Protein Data Bank (PDB) [51]. Specifically, the 3D models with PDB code 3E7A [52], 3E7B [52], and 3EGG [53] were taken into consideration because they showed a resolution lower than 2 Å, with no mutations and Homo sapiens as the origin. We also included the 3D structure with PDB code 4XPN since it is the only model reporting detailed structural and functional analyses of the GADD34:PP1 holoenzyme and its recruitment of eIF2α [5].

For each selected PDB entry, the Protein Preparation Wizard tool [55,56] was employed to add hydrogen atoms, to assign partial charges, to build missing atoms, side chains and loops. All water molecules were removed, and OPLS_2005 [57] was selected as the force field. To have a reference in the analysis of the docking results, we collected active compounds able to bind the RVxF domain of PP1 from the literature (Figure S1). Thus, The 3D structures of all active compounds and SIX2G were drawn by means Maestro GUI [58] and LigPrep tool was used for modelling and calculating their protonation state at pH 7.4 and all stereoisomers [59]. Docking simulations were performed through the application of the Glide software version 7.8 from the Schrodinger suite, [Glide, Schrçdinger, LLC, New York, NY, 2018.] by considering ligands as flexible structures and treating the receptor as rigid. The energy grid for all PDB models was built setting its outer box size to 27000 Å3 and centering the docking box on RVxF binding groove of PP1, characterized by a deep hydrophobic pocket formed by PP1 residues I169, L243, F257, R261, V264, L266, M283, L289, C291 and F293. The scaling factor of the van der Waals radii for all models was set to 1.0. The binding affinity (G-Score) for each compounds against the four target sequences was predicted using the Glide Standard Precision (SP) protocol.

Ten poses per ligand were taken into account and the default docking scoring function was used for selecting the best binding mode for each ligand. All the best complexes of SIX2G were further submitted to the calculation of the Molecular Mechanics/Generalized Born Surface Area free energy of binding (MM-GBSA ΔGbind) implemented in Prime [60,61].

References

21. Spano V, Pennati M, Parrino B, Carbone A, Montalbano A, Lopergolo A, et al. [1,2]Oxazolo[5,4-e]isoindoles as promising tubulin polymerization inhibitors. European journal of medicinal chemistry. 2016;124:840-51.

51. Velankar, S.; Burley, S.K.; Kurisu, G.; Hoch, J.C.; Markley, J.L. The Protein Data Bank Archive. *Methods in molecular biology* **2021**, *2305*, 3-21, doi:10.1007/978-1-0716-1406-8_1.

52. Kelker, M.S.; Page, R.; Peti, W. Crystal structures of protein phosphatase-1 bound to nodularin-R and tautomycin: a novel scaffold for structure-based drug design of serine/threonine phosphatase inhibitors. *Journal of molecular biology* **2009**, *385*, 11-21, doi:10.1016/j.jmb.2008.10.053.

53. Ragusa, M.J.; Dancheck, B.; Critton, D.A.; Nairn, A.C.; Page, R.; Peti, W. Spinophilin directs protein phosphatase 1 specificity by blocking substrate binding sites. *Nature structural & molecular biology* **2010**, *17*, 459-464, doi:10.1038/nsmb.1786.

54. Choy, M.S.; Yusoff, P.; Lee, I.C.; Newton, J.C.; Goh, C.W.; Page, R.; Shenolikar, S.; Peti, W. Structural and Functional Analysis of the GADD34:PP1 eIF2alpha Phosphatase. *Cell reports* **2015**, *11*, 1885-1891, doi:10.1016/j.celrep.2015.05.043.

55. Schrödinger. Schrödinger Suites. **2018**.

56. Schrödinger. Protein Preparation Wizard. *LLC: New York* **2018**.

57. Shivakumar, D.; Harder, E.; Damm, W.; Friesner, R.A.; Sherman, W. Improving the Prediction of Absolute Solvation Free Energies Using the Next Generation OPLS Force Field. *Journal of chemical theory and computation* **2012**, *8*, 2553-2558, doi:10.1021/ct300203w.

58. Schrödinger. Maestro. *LLC: New York* **2018**.

59. Schrödinger. LigPrep. *LLC: New York* **2018**.

60. Schrödinger. Prime. *LLC:New York* **2018**.

61. Maruca A, A.F., Lupia A, Romeo I, Rocca R, Moraca F, Talarico C, Bagetta D, Catalano R, Costa G, Artese A and Alcaro S. Computer-based techniques for lead identification and optimization I: Basics. *Physical Sciences Reviews* **2019**, doi:https://doi.org/10.1515/psr-2018-0113.
