# Supplementary material for: The New Microtubule-Targeting Agent SIX2G Induces Immunogenic Cell Death in Multiple Myeloma
Source: Int J Mol Sci. 2022 Sep 6;23(18):10222. doi: 10.3390/ijms231810222 (PMC9499408; doi:10.3390/ijms231810222)
Supplement: Supplementary file 1 [file ijms-23-10222-s001.zip › ijms-1875338-SI/Supplementary Table S1.pdf]

Supplementary Table S1

	3E7A		3E7B		4XPN		3EGG	
**Compound**	**Chain A**	**Chain B**	**Chain A**	**Chain B**	**Chain A**	**Chain B**	**Chain A**	**Chain B**
C31 (R)	-3.99	-4.25	-3.81	-3.12	-4.21	-4.56	-4.05	-3.80
C31 (S)	-4.03	-3.95	-3.66	-3.17	-4.06	-3.93	-4.38	-4.58
1E7-03 (R)	-3.44	-4.08	-3.01	-2.98	-3.81	-3.15	-4.08	-3.70
1E7-03 (S)	-3.74	-3.49	-3.52	-3.28	-3.53	-3.03	-4.09	-3.64
1H4	-3.16	-3.79	-3.01	-3.85	-3.77	-3.41	-3.60	-3.84
1E7-07 (R)	-3.29	-3.51	-2.89	-2.89	-3.68	-3.57	-3.22	-3.33
1E7-07 (S)	-3.50	-3.86	-3.36	-3.41	-3.57	-3.43	-3.55	-3.50
SIX2G	-3.90	-3.80	-3.19	-3.17	-4.03	-4.10	-4.03	-4.24
Average cut-off	**-3.59**	**-3.85**	**-3.32**	**-3.24**	**-3.80**	**-3.58**	**-3.85**	**-3.77**

Table S1. G-score values calculated for all active compounds and our compound SIX2G complexed with the different 3D structures of the protein PP1. For each model, the value of the average *cut-off* was also reported. All G-score values are expressed as kcal/mol.
